# Gut microbiota dynamics in a 1-year follow-up after adult liver transplantation in Northeast China

**DOI:** 10.3389/fphys.2023.1266635

**Published:** 2023-12-22

**Authors:** Ruoyan Zhang, Wei Qiu, Xiaodong Sun, Jing Li, Xiaochen Geng, Shichao Yu, Ying Liu, Heyu Huang, Mingyue Li, Zhongqi Fan, Mingqian Li, Guoyue Lv

**Affiliations:** ^1^ Department of Hepatobiliary and Pancreatic Surgery I, General Surgery Center, The First Hospital of Jilin University, Changchun, Jilin, China; ^2^ The First Hospital of Jilin University, Jilin University, Changchun, Jilin, China

**Keywords:** gut microbiota, 16S rRNA gene sequencing, liver disease, liver transplantation, dysbiosis

## Abstract

**Background:** Liver transplantation (LTx) is the most effective treatment for end-stage liver diseases. Gut microorganisms influence the host physiology. We aim to profile the dynamics of gut microbiota in the perioperative period and a 1-year follow-up of LTx recipients in Northeast China.

**Methods:** A total of 257 fecal samples were longitudinally collected from 85 LTx patients using anal swabs from pre-LTx to 1-year post-LTx. A total of 48 fecal samples from end-stage liver disease patients without LTx served as the control. 16S rRNA sequencing was used to analyze gut microbiota diversity, bacterial genera, phenotype classification, and metabolic pathways.

**Results:** The diversity of gut microbiota decreased significantly after transplantation, accompanied by a profound change in the microbial structure, which is characterized by increased abundance of facultative anaerobic bacteria dominated by g_*Enterococcus* and reduced anaerobic bacteria composition. Predicted functional analysis also revealed disturbances in the metabolic pathway of the gut microbiota. After LTx, the diversity of microbiota gradually recovered but to a less preoperative level after 1 year of recovery. Compared with pre-transplantation, the microbiome structure was characterized by an increase in *Acidaminococcus* and *Acidithiobacillus* after 1 year of transplantation.

**Conclusion:** LTx and perioperative treatment triggered gut microbial dysbiosis. The gut microbiota was restructured after LTx to near to but significantly differed from that of pre-LTx.

## Introduction

Approximately 1.3 billion people globally are affected by chronic liver disease, with approximately 2 million deaths each year attributed to liver disease, including cirrhosis, viral hepatitis, and hepatocellular carcinoma (GBD, 2017 Cirrhosis Collaborators, 2020; Asrani et al., 2019). Asia is one of the regions with the highest incidence of liver disease, with over 447 million chronic liver disease patients in China alone, increasing at a rate of 0.5% annually (Li et al., 2020). Liver transplantation (LTx) is currently recognized as the best treatment for end-stage liver disease. However, the lifespan of LTx recipients is still reduced compared with the healthy population (Lodhi et al., 2011).

The microbiota impacts the host physiology in both health and disease. The increasing focus on the human gut microbiota research provides a new perspective to deepen our understanding of the pathophysiological processes after LTx. The liver’s physiological and pathological activities are influenced by the gut microbiota. However, research on gut microbiota in LTx recipients is still insufficient, with early studies often having small sample sizes and short study periods. Meanwhile, the profile of human gut microbiota is obviously influenced by race, diet, and region. Research on gut microbiota of LTx recipients in Northeast China is still lacking.

In this study, a longitudinal analysis was conducted on 257 gut microbiota samples from 85 adult liver transplant recipients, collected at various time points, including pre-LTx, post-LTx at days 7–21, and postoperative follow-up period between 1 and 3 months and 6 and 12 months. We comprehensively investigated the diversity, relative abundance, compositional variation, phenotype, and metabolic function of the gut microbiota and profiled the dynamics of gut microbiota recovery during LTx.

## Materials and methods

### Study cohort and sample collection

This study consecutively included 85 adult patients who underwent LTx at the Liver Transplantation Center of the First Hospital of Jilin University from 1 June 2021 to 31 March 2023. The exclusion criteria of LTx patients were as follows: 1. bloody stools; 2. severe diarrhea or constipation; 3. nontolerant to sample collection; 4. antibiotic use history within 1 month before surgery; 5. combined organ failure; 6. a history of organ transplantation other than LTx; 7. combined transplantation; 8. irregular follow-up within the first year. All LTx recipients received routine immunosuppressive therapy with tacrolimus, sirolimus, or cyclosporine. Methylprednisolone and antibiotics were routinely used after surgery. A total of 48 end-stage liver disease patients without LTx were enrolled as control (C group). All the control patients received antibiotic treatment during hospitalization. The antibiotics used in the C group were similar to those administered after LTx, especially cephalosporins. Carbapenems were used for severe infections. All the details and procedures involved in the study design were in accordance with the Declaration of Helsinki. Written informed consent was obtained from all participants before sample collection. The experimental protocol was approved by the Ethics Committee of the First Hospital of Jilin University (2021–539).

A total of 305 rectal swab samples were collected from all the subjects. Among these, 257 samples were collected from the LTx group, which were divided into four groups according to the sampling time point: before the LTx sample as the Pre group (80 samples), after LTx at discharge as the Post1 group (73 samples), the postoperative follow-up period between 1 and 3 months as the Post2 group (64 samples), and the postoperative follow-up period between 6 and 12 month as the Post3 group (40 samples). In the C group, 48 samples were collected before discharge.

Stool sample collection procedure: before collection, the perianal skin was cleaned with sterile saline and medical alcohol cotton pads, and a disposable sterile rectal swab was used to collect the sample by inserting it 5 cm into the rectum through the anus and rotating it repeatedly to ensure sufficient collection. To ensure the collection effect, each sample was collected in triplicate. Sampling was performed using standard protocols to ensure the sterile collection process. The collected samples were labeled, sealed, and immediately stored at −80°C until use.

### Sampling and DNA extraction

The head of the rectal swab was repeatedly rotated and agitated in the pre-cooled saline solution. The sample was then centrifuged at ≥13,000 × g at 4°C for 10 min. The supernatant was discarded and stored on ice. DNA was extracted from the samples using the E.Z.N.A. Stool DNA Kit (Omega Bio-Tek, Inc., catalog D4015-02), according to the manufacturer’s instructions. The extracted nucleic acid concentrations were measured using the NanoDrop spectrophotometer, and the samples were stored at −80°C.

### 16S rRNA gene amplicon sequencing

For sequencing, isolated fecal DNA was used as a template for amplification, and the V3–V4 regions of 16S rRNA were amplified by performing PCR assays using the universal bacterial primer sets 341F (5′-CCTACGGGNGGCWGCAG-3′) and 805R (5′-GACTACHVGGGTATCTAATCC-3′). The PCR reaction was performed in triplicate with a 25 μL mixture containing 25 ng of template DNA, 12.5 μL PCR PreMix, 2.5 μL of each primer, and PCR-grade water to adjust the volume. The reaction steps were as follows: 98°C for 30 s; 32 cycles of denaturation at 98°C for 10 s, annealing at 54°C for 30 s, and extension at 72°C for 45 s; and then final extension at 72°C for 10 min. The PCR products were confirmed with 2% agarose gel electrophoresis. Throughout the DNA extraction process, ultrapure water, instead of a sample solution, was used to exclude the possibility of false-positive PCR results as a negative control. The PCR products were purified using AMPure XT beads (Beckman Coulter Genomics, Danvers, MA, United States) and quantified by Qubit (Invitrogen, United States). The amplicon pools were prepared for sequencing, and the size and quantity of the amplicon library were assessed on the Agilent 2100 Bioanalyzer (Agilent, United States) and with the Library Quantification Kit for Illumina (Kapa Biosciences, Woburn, MA, United States), respectively. The libraries were sequenced on the NovaSeq 6000 PE250 platform at LC-Bio Technology Co., Ltd. (Hangzhou, China).

### Bioinformatics analysis and statistical processing

Analysis of paired-end data obtained by sequencing requires the following steps: 1) splitting data and removing primer and barcode sequences; 2) splicing and filtering data (cutadapt-v1.9, FLASH-v1.2.8, fqtrim-v0.94, and vsearch-v2.3.4); 3) denoising to obtain the feature sequence and abundance table (DADA2-v2019.7); 4) evaluating alpha and beta diversity (QIIME2-v2019.7); 5) species annotations (SILVA-Release138, NT-16S database, and confidence threshold for annotations: 0.7); 6) analyzing differences between comparison groups; 7) function prediction (PICRUSt-v2.2.0b, KEGG database, and MetaCyc database); 8) phenotype prediction (https://github.com/knights-lab/BugBase); 9) longitudinal analysis (q2-longitudinal); 10) correlation heatmap generation (R-3.5.2-corrplot). R (v3.5.2) packages such as ade4-v1.7.13, ggplot2-v3.2.0, and vega-v2.5.4 were mainly used for plotting the graphs. The Mann–Whitney U test was used to compare the differences between the two groups of samples with biological replicates. The Kruskal–Wallis test was used to compare the differences between the multiple groups of samples with biological replicates. Continuous correction chi-squared analysis was used to compare categorical variables. *p*-value < 0.05 was considered statistically significant.

## Results

### Demographic characteristics of patients

In the LTx group, 83 recipients underwent DCD LTx, one underwent living donor LTx, and one underwent DCD split LTx (right half). A total of 84 recipients underwent the first transplantation, and one recipient underwent the second transplantation. The median age (range) was 50 (29–71) years, with 25 female (29.4%) and 60 male (70.6%) subjects. The median body mass index (BMI) (range) before the operation was 24.5 (16.5–36.5) kg/m^2^. Primary diseases of all enrolled patients are as follows: 48 cases of hepatitis B, 4 cases of hepatitis C, 11 cases of alcoholic liver disease, 4 cases of drug-induced liver injury, 8 cases of primary biliary cirrhosis, 2 cases of autoimmune liver disease, and 8 cases of unknown etiology. Among the enrolled patients, 35 cases (41.2%) had liver cancer, including 29 cases of hepatitis B, 3 cases of hepatitis C, 2 cases of alcoholic liver disease, and 1 case of primary biliary cirrhosis. The patients were followed up until 31 March 2023, with an overall survival rate of 96.5%.

The control group showed comparable demographic characteristics as the LTx group: the median age (range) was 52 (27–66) years, with 14 female (29.2%) and 34 male (70.8%) subjects. The median body mass index (BMI) (range) was 23.2 (14.6–35.6) kg/m^2^. Primary diseases were hepatitis B (23 cases), hepatitis C (1 case), alcoholic liver disease (14 cases), drug-induced liver injury (6 cases), primary biliary cirrhosis (2 cases), and unknown etiology (2 cases). Among the C group, 12 cases (25%) had liver cancer, including 9 cases of hepatitis B, 1 case of hepatitis C, and 2 cases of alcoholic liver disease.

Table 1 summarizes the baseline characteristics of these patients. Clinical information is shown in Table 2. Details of clinical measures for individual samples are provided in Supplementary Table S1.

**TABLE 1:** Demographic and clinical characterization of LTx and the C group^a^.

Parameter	Characteristic or result	Values for the indicated group		*p-value*
		LTx (*n = 85*)	C (*n = 48*)	
Demographic characteristics	Number of male subjects (%)	62 (72.9)	34 (70.8)	0.963
	Median age (years) (range)	50 (29–71)	52 (27–66)	0.974
	BMI (kg/m^2^; median, range)	24.5 (16.5–36.5)	23.2 (14.6–35.6)	0.130
Laboratory results (median and range)	ALT (U/L)	23.1 (7.9–133.0)	28.8 (1.1–136.2)	0.108
	AST (U/L)	36.1 (15.5–188.7)	50.6 (12.0–423.1)	0.003
	GGT (U/L)	37.7 (10.3–619.0)	43.1 (11.3–421.9)	0.026
	ALP (U/L)	117.9 (51.6–1,237.4)	117.8 (38.4–682.6)	0.577
	CHE (U/L)	3,052 (1,246–10457)	2,993 (709–7,396)	0.315
	ALB (g/L)	33.3 (19.1–50.7)	32.5 (26.2–39.4)	0.173
	TB (mg/dL)	2.37 (0.50–40.97)	7.93 (0.96–38.25)	<0.001
	IDB (mg/dL)	1.61 (0.39–17.02)	3.43 (0.61–21.63)	<0.001
	INR	1.35 (0.91–2.84)	1.56 (1.04–4.27)	<0.001
	Glu (mmol/L)	5.95 (4.19–24.23)	6.53 (4.52–23.61)	0.026
	Cr (mg/dL)	0.71 (0.26–3.12)	0.68 (0.300–3.21)	0.553
	Hb (g/L)	102.0 (55–177)	92.5 (55–168)	0.019
	MELD-Na	14 (6–51)	22 (9–52)	<0.001
Cirrhosis etiology (%)	HBV	48 (56.5)	23 (47.9)	0.084
	HCV	4 (4.7)	1 (2.1)	
	ALD	11 (12.9)	14 (29.2)	
	DILI	4 (4.7)	6 (12.5)	
	PBC	8 (9.4)	2 (4.2)	
	AILD	2 (2.4)	0 (0.0)	
	Unknown	8 (9.4)	2 (4.2)	

Note: ^a^Continuous variables were compared using the Kruskal–Wallis test for comparisons between patient groups. Continuous correction chi-squared analysis was used to compare categorical variables, with *p* < 0.01.

**TABLE 2:** Demographic and clinical characterization of study groups^a^.

Parameter	Characteristic or result	Values for the indicated group					*p-value* ^b^
		Total (*n = 85*)	Pre (*n = 80*)	Post1 (*n = 73*)	Post2 (*n = 64*)	Post3 (*n = 40*)	
Demographic characteristics	Number of male subjects (%)	62 (72.9)	59 (73.8)	52 (71.2)	48 (75.0)	29 (72.5)	0.965
	Median age (years) (range)	50.0 (29–71)	51.0 (29–71)	50.0 (29–71)	50.0 (29–71)	50.5 (33–69)	0.879
	BMI (kg/m^2^; median and range)	24.5 (16.5–36.5)	24.6 (16.5–36.5)	22.3 (15.6–32.8)	22.8 (15.7–30.9)	23.1 (16.2–30.9)	0.017
Laboratory results (median and range)	ALT (U/L)	23.1 (7.9–133.0)	23.3 (7.9–133.0)	44.4 (3.0–268.6)	18.9 (3.9–100.8)	23.1 (5.8–162.7)	<0.001
	AST (U/L)	36.1 (15.5–188.7)	36.5 (15.5–188.7)	18.0 (7.1–84.5)	22.5 (11.5–239.8)	28.1 (15.0–162.7)	<0.001
	GGT (U/L)	37.7 (10.3–619.0)	38.9 (10.3–619.0)	72.9 (28.6–649.0)	34.1 (11.4–375.0)	26.9 (8.0–409.7)	<0.001
	ALP (U/L)	117.9 (51.6–1,237.4)	119.5 (51.6–1,237.4)	84.0 (33.7–283.5)	98.7 (51.1–412.1)	109.3 (55.7–320.8)	<0.001
	CHE (U/L)	3,052 (1,246–10,457)	3,163 (1,246–10,457)	3,726 (2,148–5,848)	6,475 (3,745–10,942)	8,281 (5,119–12,713)	<0.001
	ALB (g/L)	33.3 (19.1–50.7)	33.4 (19.1–50.7)	37.5 (32.0–51.8)	42.8 (31.8–68.7)	43.9 (37.6–50.4)	<0.001
	TB (mg/dL)	2.37 (0.50–40.97)	2.37 (0.50–40.5)	1.13 (0.46–7.20)	1.10 (0.34–3.95)	0.90 (0.39–1.62)	<0.001
	IDB (mg/dL)	1.61 (0.39–17.02)	1.59 (0.39–14.99)	0.73 (0.26–3.59)	0.81 (0.28–2.31)	0.70 (0.29–1.37)	<0.001
	INR	1.35 (0.91–2.84)	1.35 (0.91–2.84)	1.05 (0.87–1.39)	—	—	<0.001
	Glu (mmol/L)	5.95 (4.19–24.23)	6.04 (4.19–24.23)	5.75 (3.49–18.52)	6.08 (4.55–12.08)	5.63 (4.37–8.63)	0.037
	Cr (mg/dL)	0.71 (0.26–3.12)	0.71 (0.26–3.12)	0.69 (0.30–1.87)	0.96 (0.50–1.86)	1.00 (0.70–1.60)	<0.001
	Hb (g/L)	102.0 (55–177)	102.5 (55–177)	97.0 (63–131)	127.5 (85–177)	143.5 (71–191)	<0.001
	MELD-Na	14 (6–32)	14 (6–32)	—	—	—	
Cirrhosis etiology (%)	HBV	48 (56.5)	44 (55.0)	39 (53.4)	37 (57.8)	24 (60.0)	0.999
	HCV	4 (4.7)	4 (5.0)	4 (5.5)	1 (1.6)	1 (2.5)	
	ALD	11 (12.9)	11 (13.8)	9 (12.3)	9 (14.1)	7 (17.5)	
	DILI	4 (4.7)	4 (5.0)	5 (6.8)	2 (3.1)	1 (2.5)	
	PBC	8 (9.4)	8 (10.0)	6 (8.2)	6 (9.4)	4 (10.0)	
	AILD	2 (2.4)	2 (2.5)	2 (2.7)	2 (3.1)	0 (0.0)	
	Unknown	8 (9.4)	7 (8.8)	7 (9.6)	7 (10.9)	3 (7.5)	

Note: ^a^Continuous variables were compared using the Kruskal–Wallis test for comparisons between patient groups. Continuous correction chi-squared analysis was used to compare categorical variables, with *p* < 0.01.

^b^All statistical analyses between groups were comparing Pre, Post1, Post2, and Post3 groups. Total was the summary of preoperative indicators of all enrolled recipients and those who did not participate in the comparison between groups.

### Quality control

We plotted the rarefaction curve using the number of sequences as the abscissa and the corresponding numbers of amplicon sequence variants (ASVs) as the ordinate (Supplementary Figure S1A). The rarefaction curve reflects the rationality of sequencing data and the richness of ASVs in the sample. In this study, the curves of each specimen were ended flat, indicating the sequencing was sufficient to detect rare species. The Pre group had the highest richness, Post1 group had the lowest richness, while the Post2 and Post3 groups were located in between. The rarefaction curve of the C group was higher than that of the Post1 group, while lower than that of the Post3 group (Supplementary Figure S1B).

A Venn diagram was used to show the distribution of ASVs obtained in each group (Supplementary Figure S1C). A total of 25,565 ASVs were obtained in this study, including 12,202 in the Pre group, 5,182 in the Post1 group, 7,822 in the Post2 group, 5,456 in the Post3 group, and 5,615 in the C group. In all samples, 28 phyla, 73 classes, 168 orders, 300 families, 799 genera, and 1,641 species were identified (Supplementary Table S3).

### Microbial diversity was reduced after LTx

**FIGURE 1:**
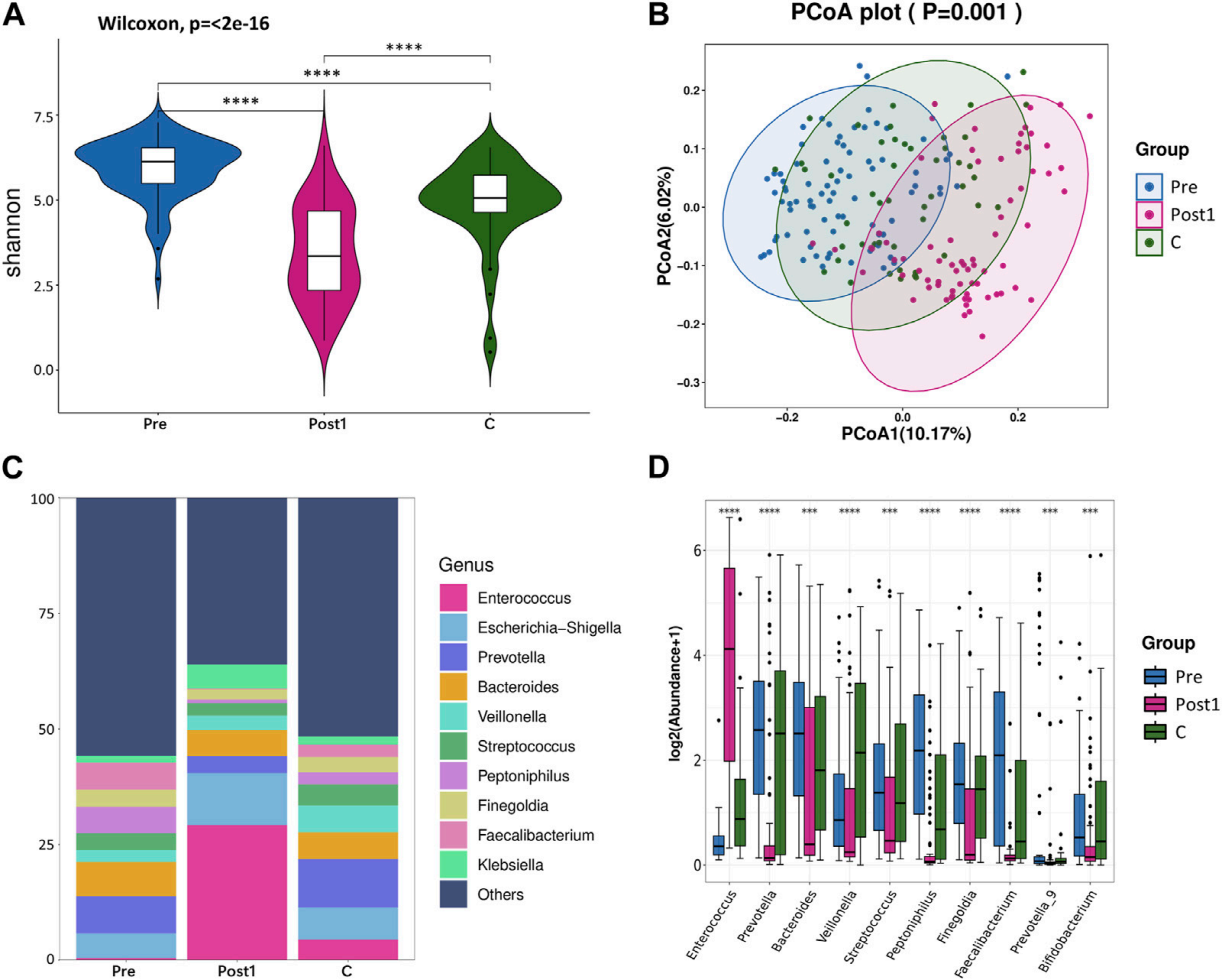
**(A)** Alpha diversity between Pre, Post1, and C groups. Comparison of the violin plot depicting the Shannon index. The Wilcoxon test was used to determine the statistical significance. **(B)** Beta diversity between the three groups. Principal coordinate analysis plot based on the unweighted UniFrac distance. The *p*-values of each plot were all less than 0.01. Statistical significance was determined by ANOSIM (analysis of similarities). **(C)** Relative abundance distribution of top 10 species at the genus level in each group. **(D)** Differential abundance testing. Here, 10 species with statistically significant differences at the genus level are shown in the order of relative abundance from high to low. The Mann–Whitney U test was used to determine the statistical significance. *p < 0.05, **p < 0.01, ***p < 0.001, and ****p < 0.0001.

Antibiotics were commonly administered after LTx to prevent and treat postoperative infections. To distinguish the respective impacts of postoperative antibiotic usage and LTx on gut microbiota, the microbial profiles of the pre, Post1, and C groups were compared (Figure 1). Notable differences in microbial diversity were observed among the three groups (Figures 1A, B). The Shannon index was highest in the pre-group, followed by C and Post1 groups. The top 10 genera in each group are shown in Figure 1C, with the most notable change being the relative abundance of g_*Enterococcus* across all three groups. The top 10 taxa with statistically significant differences in relative abundance were highlighted (Figure 1D). Notably, g_*Enterococcus* had the highest relative abundance in the Post1 group, while g_*Veillonella* had the highest abundance in the C group. The other eight genera in the graph all had the highest relative abundance in the Pre group.

**FIGURE 2:**
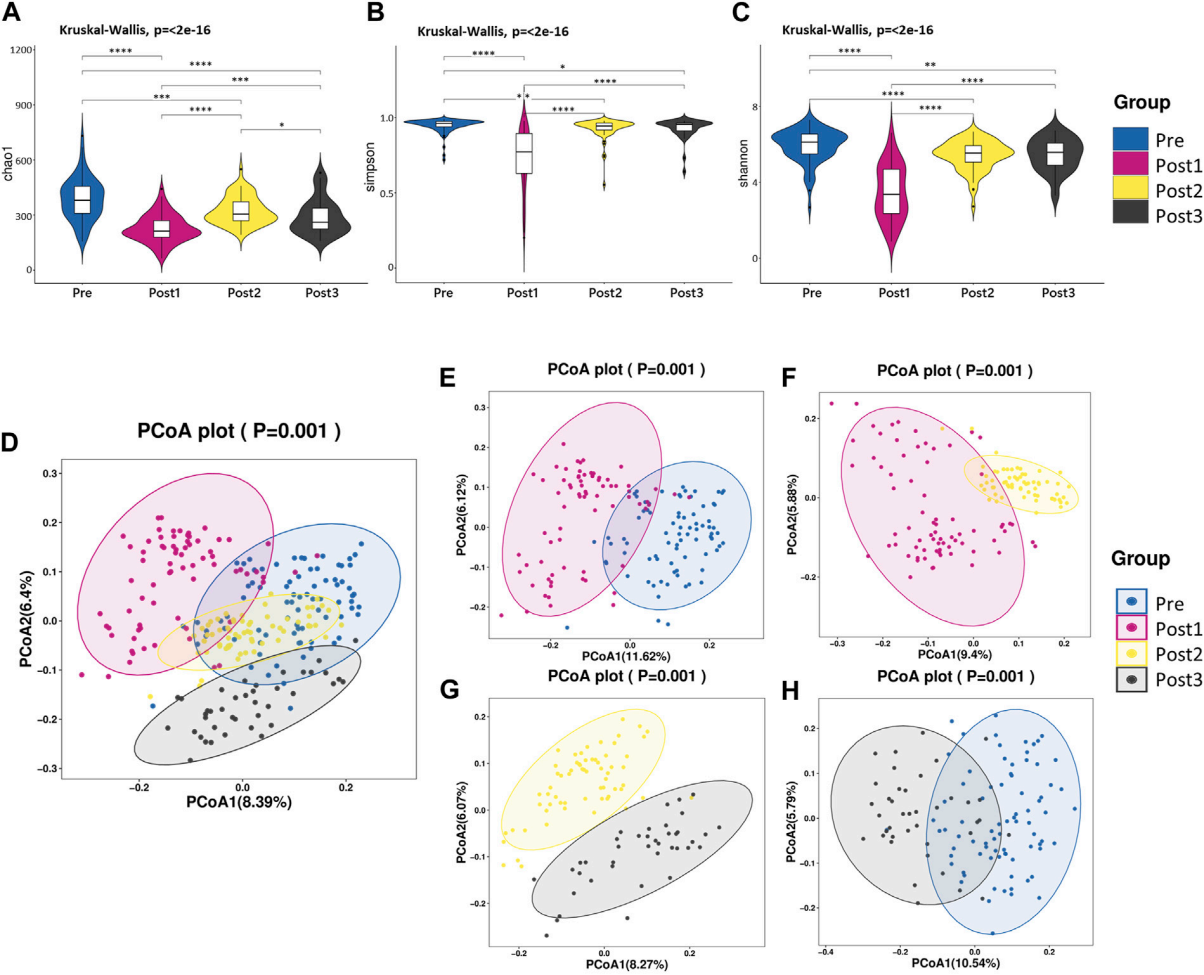
Diversity analysis. **(A–C)** Alpha diversity. Comparison of the violin plot depicting the Chao1 index **(A)**, Simpson index **(B)**, and Shannon index **(C)**. The Kruskal–Wallis test was used to determine the statistical significance. *p < 0.05, **p < 0.01, ***p < 0.001, and ****p < 0.0001. **(D–H)** Beta diversity. Principal coordinate analysis plot based on unweighted UniFrac distance. Each point in the figure represents a sample, and different colors represent different groups. The contrast groups represented by **(D–H)** plots were Pre, Post1, Post2, and Post3 groups **(D)**; Pre and Post1**(E)**; Post1 and Post2 **(F)**; Post2 and Post3 **(G)**; Pre and Post3 **(H)**. PCo1 and PCo2 were 8.39% and 6.4% **(D)**, 11.62% and 6.12% **(E)**, 9.4% and 5.88% **(F)**, 8.27% and 6.07% **(G)**, and 10.54% and 5.79% **(H)** of the total variance on the *x*-axis and *y*-axis, respectively. The R values in each figure were 0.3957 **(D)**, 0.4185 **(E)**, 0.3892 **(F)**, 0.4092 **(G)**, and 0.4636 **(H)**. The *p*-values of each plot were all less than 0.01. Statistical significance was determined by ANOSIM.

We compared the alpha diversity of Pre, Post1, Post2, and Post3 groups using the Chao1 index, Simpson index, and Shannon index (Figures 2A–C). Significant differences in bacterial richness and diversity were observed among the four groups (*p <* 0.05). The highest bacterial richness and diversity were found in the Pre group, while the lowest was found in the Post1 group, determined by all the three indexes, indicating that surgery and perioperative treatment significantly affected the microbiome structure. The alpha diversity recovered after 1–3 months subsequently but remained lower than that of the Pre group. The alpha diversity did not continue to recover in the first year to a comparable level as pre-LTx and was even significantly reduced compared to the former time point, according to the Chao1 index (Figure 2A). The Simpson index and Shannon index showed similar results as the Chao1 index, except no significant differences were found between the Post2 and Post3 groups (Figures 2B, C). Overall, the alpha diversity decreased significantly in the early postoperative period and rapidly recovered after LTx. However, the alpha diversity of the recipient’s gut microbiota cannot be fully recovered to the preoperative level in the first year after LTx.

The beta diversity differences were compared among these four groups using PCoA based on unweighted UniFrac distance (Figures 2D–H). According to PCoA among the four groups, PCo1 was 8.39% and PCo2 was 6.4% (Figure 2D). Similar to the alpha diversity, the Post1 group was segregated from other groups, suggesting the gut microbiota composition was profoundly affected. We further compared two neighboring time points’ beta diversity, and they all exhibited significant differences (Figures 2E–G), in line with the alpha diversity results. When we compared the pre-LTx gut microbiota composition with that after 6–12 months after LTx, we found that the bacterial structure was not recovered to the preoperative level (Figure 2H).

This result reflected that LTx and perioperative treatment triggered dysbiosis, which recovered shortly after transplantation; however, the diversity and composition of the gut microbiota were different from that before transplantation throughout the first year after LTx.

### Bacterial compositions and cluster analysis of LTx recipients’ gut microbiota

**FIGURE 3:**
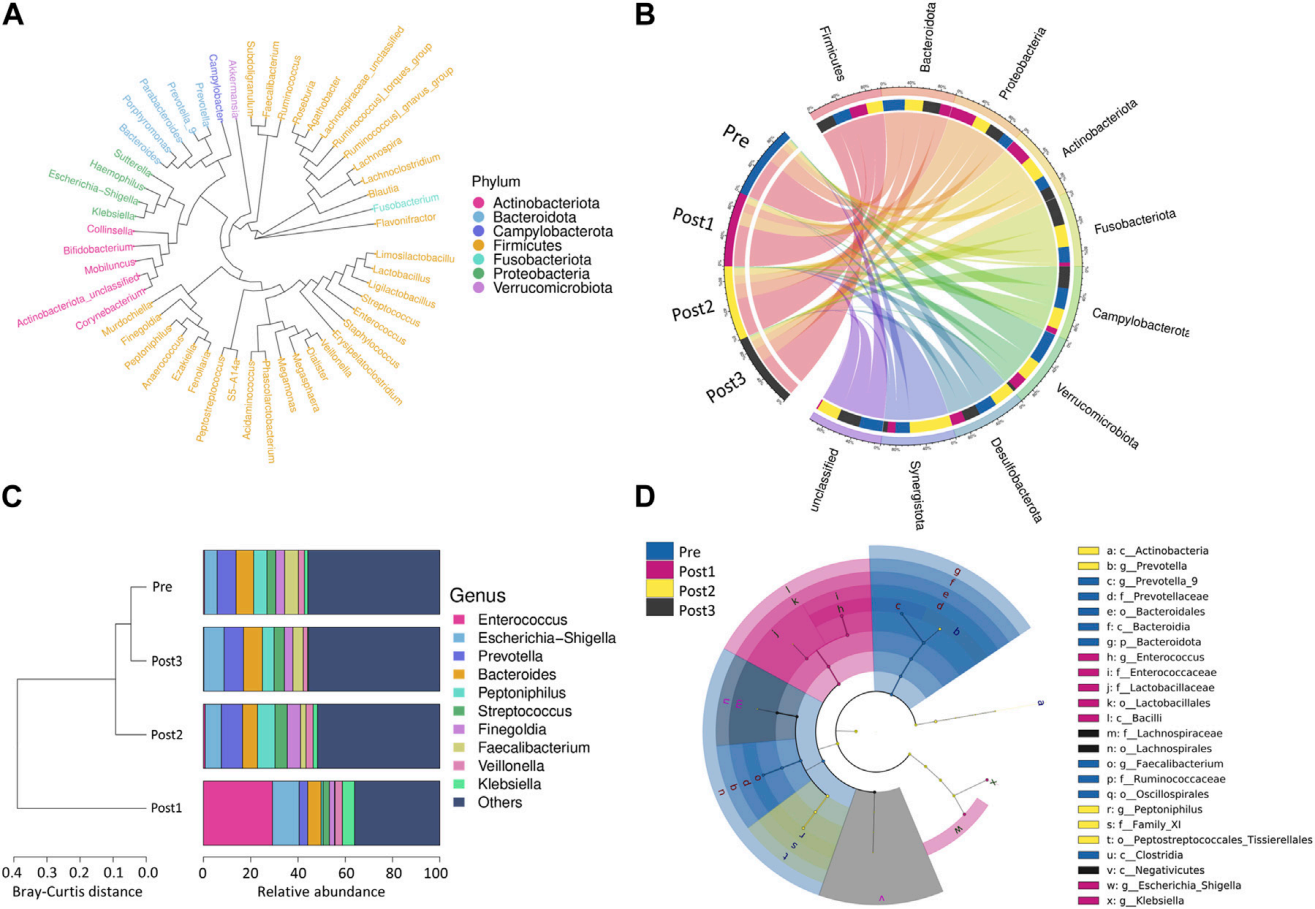
Bacterial compositions and cluster analysis. **(A)** Evolutionary tree of the top 50 microbial taxa identified in all samples. The different colors represent the phyla. Taxa that were further apart from each other on the tree were more distantly related. **(B)** Taxonomy abundance Circos showed the 10 most abundant bacteria at the phylum level and their proportion in each group. **(C)** Cluster analysis: the left side of the figure shows the Bray–Curtis distance clustering tree structure, while the right side of the figure shows the relative abundance distribution of top 10 species at the genus level in each group. **(D)** LEfSe analysis shows the branching evolutionary relationships of gut microbes that differed among the groups.

The cladogram shows the evolutionary tree of the top 50 microbial taxa identified in all samples (Figure 3A), where taxa that were further apart from each other on the tree diverged earlier from a common ancestor. Taxonomy abundance Circos showed the 10 dominant bacteria at the phylum level and their proportion in each group (Figure 3B). Despite the similar constitution of the top five bacteria in all groups, namely, Firmicutes, Bacteroidetes, Proteobacteria, Actinobacteria, and Fusobacteria, the ranks of Proteobacteria and Bacteroidetes were reversed in the Post1 group. Details of the proportion of each phylum in each group and the statistical differences at the level of phyla are provided in Table 3; Supplementary Figure S2.

**TABLE 3:** Proportion of each phylum in each group.

Phylum	Proportion of each group (%)				Mean value (%)
	Pre	Post1	Post2	Post3	
Firmicutes	58.03	57.90	54.07	60.10	57.52
Bacteroidota	24.70	12.74	20.94	19.39	19.44
Proteobacteria	8.87	19.12	12.07	11.93	13.00
Actinobacteriota	4.89	9.07	9.03	4.82	6.95
Fusobacteriota	1.33	0.30	1.89	2.33	1.46
Campylobacterota	0.96	0.26	0.94	0.99	0.79
Verrucomicrobiota	0.81	0.34	0.52	0.08	0.44
Desulfobacterota	0.27	0.20	0.31	0.23	0.25
Synergistota	0.05	0.03	0.13	0.01	0.06
Unclassified	0.06	0.00	0.05	0.05	0.04
Total	99.96	99.94	99.95	99.96	99.95

Cluster analysis displays the similarity between groups and the relative abundance of species at the genus level in each group (Figure 3C). The left side of the figure shows the Bray–Curtis distance clustering tree structure, which then shows that the Post1 group had the lowest similarity with the other three groups, while the Pre group and the Post2 and Post3 groups shared similarity. The right side of the figure shows the relative abundance distribution of species at the genus level in each group, with the proportion of the top 10 ranked genera in each group. Among all the samples, the top 10 dominant genera were g_*Enterococcus*, g_*Escherichia*−*Shigella*, g_*Prevotella*, g_*Bacteroides*, g_*Peptoniphilus*, g_*Streptococcus*, g_*Finegoldia*, g_*Faecalibacterium*, g_*Veillonella*, and g_*Klebsiella*. We found that g_*Enterococcus* was remarkably enriched in the Post1 group. These bacteria showed a unique composition in the Post1 group.

We further demonstrated the branching evolutionary relationships of gut microbes through LEfSe analysis (Figure 3D). The linear discriminant analysis (LDA) threshold was set to 4.3. A total of 24 enriched species were identified. Among them, nine species were enriched in the Pre group, seven species were enriched in the Post1 group, five species were enriched in the Post2 group, and three species were enriched in the Post3 group.

### Longitudinally comparing the differences in gut microbiota at the genus level

**FIGURE 4:**
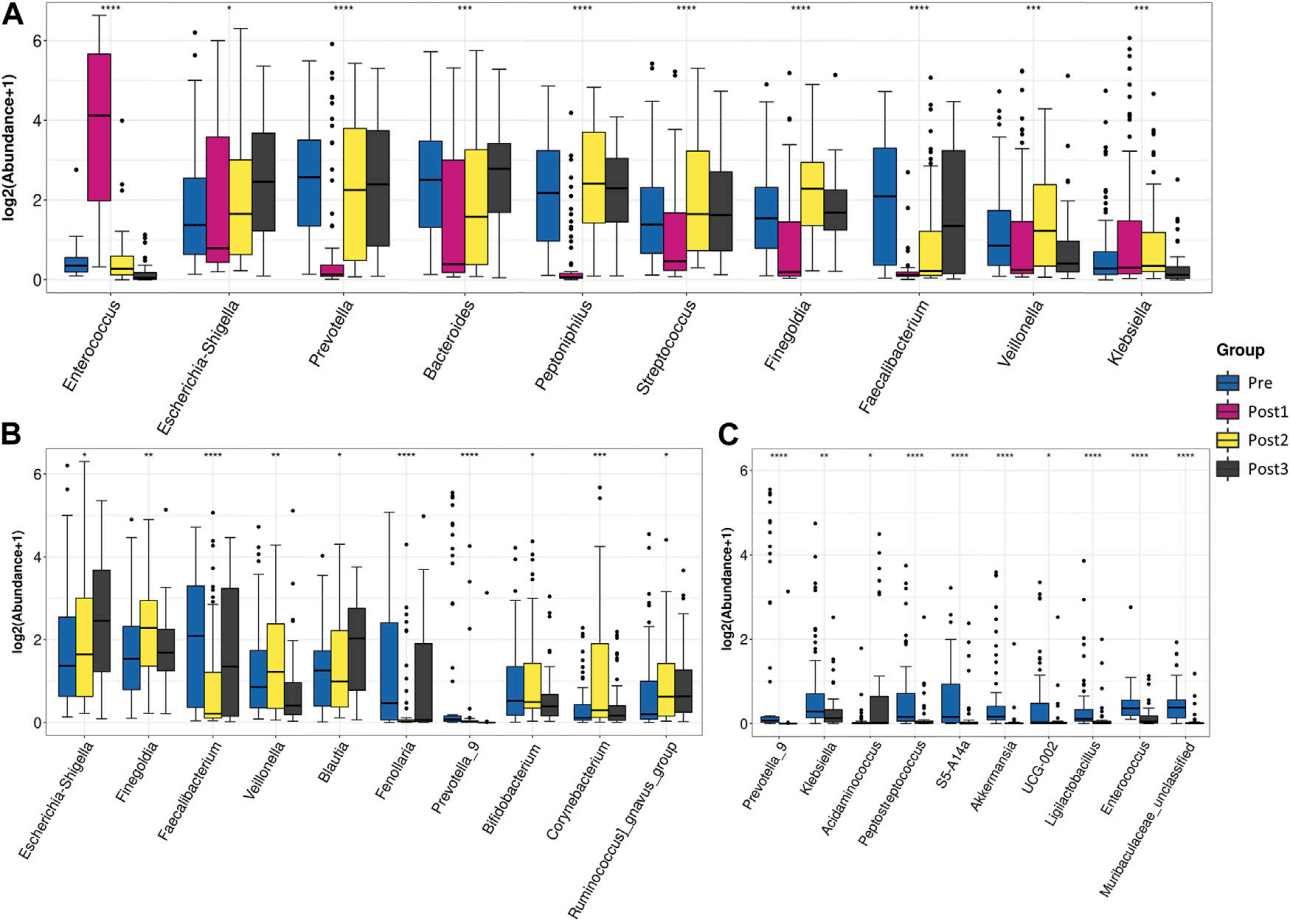
Differential abundance testing. Taxa with statistically significant differences in relative abundance were shown. The contrast groups represented by **(A–C)** plots were Pre, Post1, Post2, and Post3 ; Pre, Post2, and Post3 **(B)**; Pre and post3 **(C)**. Each plot presented the 10 species with statistically significant differences at the genus level in the order of relative abundance from high to low. The Kruskal–Wallis test was used to determine the statistical significance. *p < 0.05, **p < 0.01, ***p < 0.001, and ****p < 0.0001.

To further elucidate the differences in species composition among groups, we conducted differential abundance testing at the genus level (Figures 4A–C). Compared with other time points, the surgery significantly triggered upregulation of g_*Enterococcus*, g_*Actinobacteriota*, and g_*Ligilactobacillus*, and triggered the decline of the relative abundance of most gut microbiota, including g_*Prevotella*, g_*Peptoniphilus*, g_*Streptococcus*, g_*Finegoldia*, g_*Veillonella*, g_*Faecalibacterium*, g_*Anaerococcus*, and g_*Dialister* (Figure 4A).

To investigate the difference between pre- and post-groups, we excluded the Post1 group from the analysis (Figure 4B). The top 10 differentially abundant genera among the Pre, Post2, and Post3 groups were g_*Escherichia*–*Shigella*, g_*Finegoldia*, g_*Faecalibacterium*, g_*Veillonella*, g_*Blautia*, g_*Fenollaria*, g_*Prevotella*_9, g_*Bifidobacterium*, g_*Corynebacterium*, and g_*Ruminococcus*_*gnavus*_group. The gut microbiota after 1–3 months of recovery from LTx was distinguished from counterparts of the preoperative period and 6–12 months of the postoperative period by a high proportion of g_*Finegoldia*, g_*Veillonella*, and g_*Corynebacterium*, and a low proportion of g_*Faecalibacterium* and g_*Fenollaria*. After 6–12 months of recovery, the feature of short recovery was disappeared as g_*Finegoldia*, g_*Veillonella*, and g_*Corynebacterium* decreased and g_*Faecalibacterium* and g_*Fenollaria* increased to comparable levels of the preoperative state.

When we compared the Pre group with the Post3 group (Figure 4C), we found that the top 10 differentially abundant genera were those that were highly composed in the Pre group, with an exception of g_*Acidaminococcus*, the proportion of which was higher in the Post3 group. We found that the bacteria that had significant changes in the Post1 group had recovered, indicating that the impact of the Post1 stage on gut microbiota was not permanent.

In this study, the ratio of statistically significant differences among the top 30 genera was 29:30, when comparing the four groups together. When the Post1 group was excluded from the analysis, this ratio became 15:30. The ratio decreased to 5:30 and 3:30 when comparing the Pre group with Post2 and Post3 groups, respectively (Table 4). With the extension of follow-up time, the gap of the gut microbiota structure between post- and pre-samples was narrowed.

**TABLE 4:** Statistical differences of the top 30 bacterial genera in different comparison groups.

	Group of contrasts			
	Pre vs. Post1 vs. Post2 vs. Post3	Pre vs. Post2 vs. Post3	Pre vs. Post2	Pre vs. Post3
Top 30 bacterial genera in relative abundance	*Enterococcus	*Prevotella*	*Prevotella*	*Prevotella*
	*Escherichia–Shigella	*Bacteroides*	*Bacteroides*	*Bacteroides*
	*Prevotella	*Escherichia–*Shigella*	*Peptoniphilus*	*Escherichia*–*Shigella*
	*Bacteroides	*Peptoniphilus*	*Escherichia*–*Shigella*	*Peptoniphilus*
	*Peptoniphilus	*Finegoldia	*Finegoldia*	*Faecalibacterium*
	*Streptococcus	*Streptococcus*	*Streptococcus*	*Streptococcus*
	*Finegoldia	*Faecalibacterium	*Faecalibacterium	*Finegoldia*
	*Faecalibacterium	*Anaerococcus*	*Porphyromonas*	*Fenollaria*
	*Veillonella	*Porphyromonas*	*Anaerococcus*	*Prevotella_9
	*Klebsiella	*Veillonella	*Veillonella*	*Anaerococcus*
	*Anaerococcus	*Dialister*	*Prevotella_9	*Blautia*
	*Porphyromonas	*Blautia	*Dialister*	*Dialister*
	*Blautia	*Fenollaria	*Blautia*	*Veillonella*
	*Dialister	*Prevotella_9	*Fenollaria	*Porphyromonas*
	*Corynebacterium	*Bifidobacterium	*Bifidobacterium*	*Lactobacillus*
	*Fenollaria	*Corynebacterium	*Corynebacterium	*Ruminococcus]_gnavus*_group
	*Prevotella_9	*Ruminococcus]*_gnavus*_group	*Klebsiella*	*Megamonas*
	*Bifidobacterium	*Fusobacterium*	*Ruminococcus*]_*gnavus*_group	*Roseburia*
	*Ruminococcus_*gnavus*_group	*Klebsiella	*Fusobacterium*	*Bifidobacterium*
	*Actinobacteriota_unclassified	*Roseburia*	*Lachnospiraceae*_unclassified	*Lachnospiraceae*_unclassified
	*Ligilactobacillus	*Lachnospiraceae*_unclassified	*Agathobacter*	*Agathobacter*
	*Fusobacterium	*Agathobacter	*Roseburia*	*Fusobacterium*
	*Lachnospiraceae_unclassified	*Megamonas*	*Ezakiella*	*Klebsiella
	*Roseburia	*Ezakiella	*Campylobacter*	*Ezakiella*
	*Megamonas*	*Lactobacillus	*Lachnoclostridium*	*Campylobacter*
	*Lachnoclostridium	*Campylobacter*	*Peptostreptococcus*	*Subdoligranulum*
	*Parabacteroides	*Lachnoclostridium*	*Megasphaera*	*Ruminococcus*]*_torques*_group
	*Lactobacillus	*Ruminococcus_torques*_group	*Lactobacillus	*Lachnospira*
	*Agathobacter	*Megasphaera	*Haemophilus*	*Collinsella*
	*Megasphaera	*Subdoligranulum*	*Ruminococcus*]*_torques*_group	*Acidaminococcus
Ratio	99.7%	50.0%	16.7%	10.0%

Note: The symbol “*” represents the presence of a statistical difference.

### Correlation between gut microbiota and clinical indicators

**FIGURE 5:**
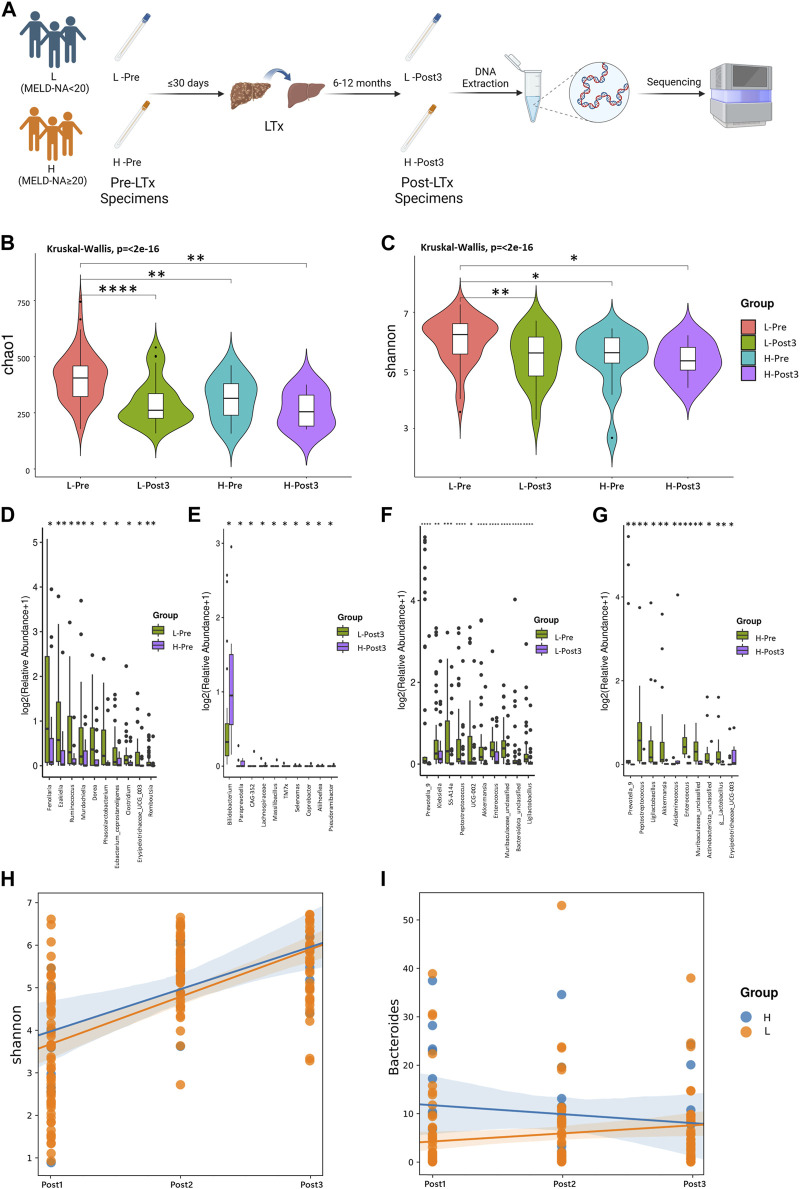
Correlation between gut microbiota and MELD-Na score. Using MELD-Na≥20 as a threshold, the Pre and Post3 groups were divided into H-Pre, L-Pre, H-Post3, and L-Post3 **(A)**. **(B, C)** Alpha diversity. Comparison of the violin plot depicting the Chao1 index **(B)** and Shannon index **(C)**. The Kruskal–Wallis test was used to determine the statistical significance. Taxa with statistically significant differences in relative abundance were shown. The contrast groups represented by **(C–F)** plots were L-Pre and H-Pre ; L-post3 and H-post3 **(E)**; L-Pre and L-Post3 **(F)**; H-Pre and H-Post3 **(G)**. Each plot presented the 10 species with statistically significant differences at the genus level in the order of relative abundance from high to low. The statistical method was the Mann-Whitney U test. *p < 0.05, **p < 0.01, ***p < 0.001, and ****p < 0.0001. Using the linear mixing model, the correlation between the MELD-Na score and the Shannon index or the bacterial genus was further analyzed. **(H)** The results showed that there was no statistically significant difference in the impact of MELD-Na on the Shannon index (*p* = 0.472). **(I)** There was a statistically significant difference in the impact of MELD-Na on g_*Bacteroides* (*p* = 0.001). The statistical method was ANOVA analysis, with *p* < 0.05.

In order to explore the relationship between disease severity and gut microbiota in LTx recipients, we grouped LTx patients based on their MELD-Na scores [MELD-Na = MELD +1.59 × (135 − Na)] before LTx. Patients with MELD-Na≥20 were selected in the H group, and patients with MELD-Na<20 were selected in the L group (Figure 5A). The alpha diversity was compared using the Chao1 index and Shannon index (Figures 5B, C). When comparing the high and low MELD-Na scores, there were statistical differences between H-Pre and L-Pre, but no significant differences were observed between H-Post3 and L-Post3. Moreover, when comparing the status before transplantation and at the end of the follow-up, there were statistical differences between L-Pre and L-Post3, but no statistical differences were observed between H-Pre and H-Post3. Differences in gut microbiota at the genus level between the groups were also observed (Figures 5D–G), and the most pronounced differences in gut microbiota were observed between L-Pre and H-Pre (Figure 5D). When comparing the Post3 group, except for g_*Bifidobacterium*, the relative abundances of differentially present species in L-Post3 and H-Post3 were generally low (Figure 5E). This suggests that the differences in gut microbiota between high and low MELD-Na patients mostly disappeared after 6–12 months. As expected, the species exhibited significant differences in both MELD groups between Pre and Post3 timepoints (Figures 5F, G), in line with the former result.

To further explore the longitudinal impact of the preoperative MELD-Na score level of LTx recipients on their postoperative gut microbiome changes, a linear mixed model was used to analyze the impact of high or low MELD-Na scores (H and L groups) on the microbial diversity and specific microorganisms. The results showed that there was no statistically significant difference in the impact of MELD-Na on the Shannon index (*p* = 0.472) (Figure 5H). The impact of high or low MELD-Na scores (H and L groups) on the relative abundance of top five microbial phyla and genera was analyzed. Among the genera and phyla evaluated, the MELD-Na scores only had a statistically significant effect on g_*Bacteroides* (*p* = 0.001) (Figure 5I). There was no statistical difference in other bacteria phyla and genera (Supplementary Figure S3).

**FIGURE 6:**
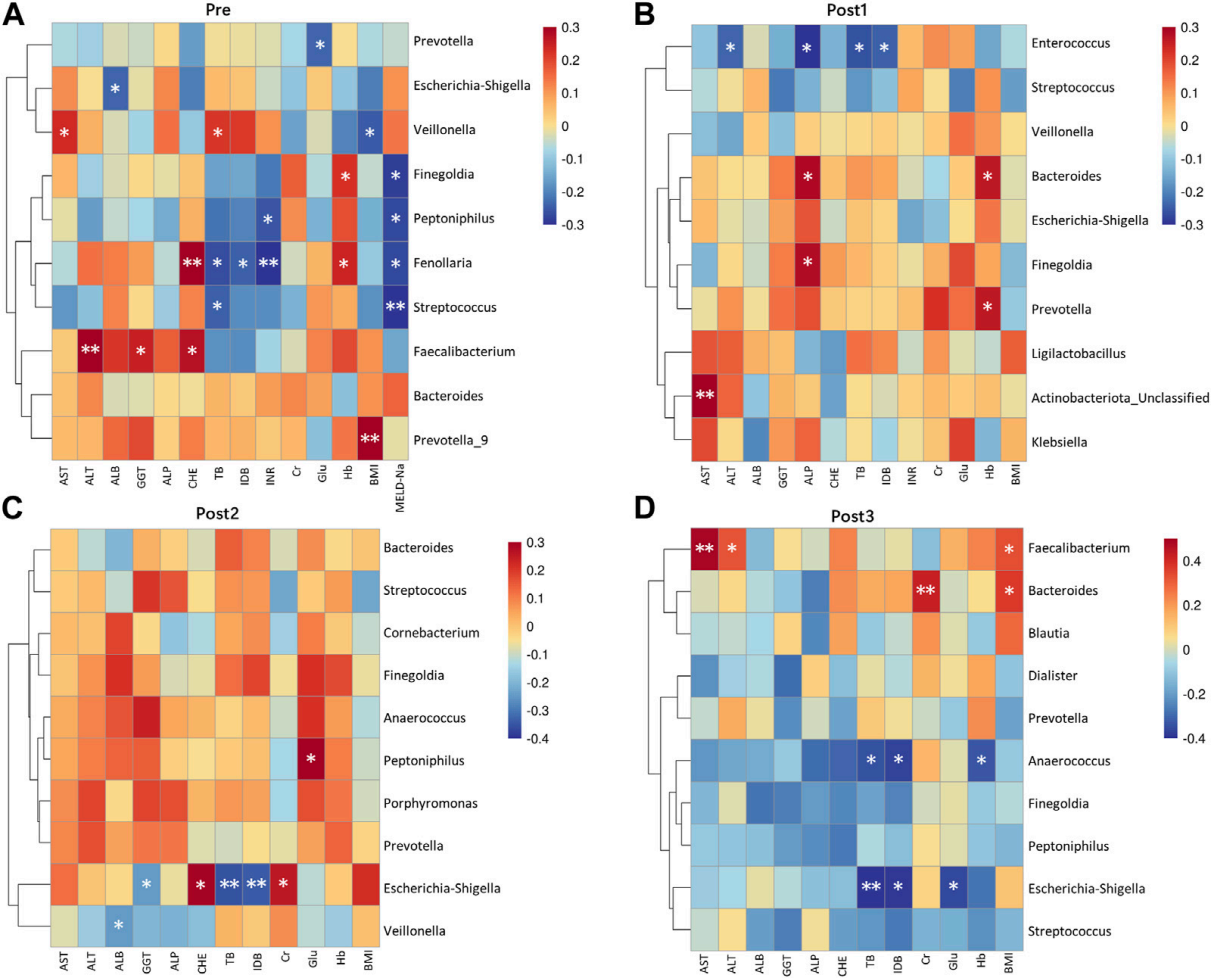
Correlation heatmap between genera and clinical indicators. Microbiota at the genus level, ranking in the top 10 relative abundances from each group, were included in the analysis alongside concurrent clinical parameters. The corresponding groups, represented by **(A–D)**, were Pre **(A)**; Post1 **(B)**; Post2 **(C)**; Post3 **(D)**. Genera were arranged from highest to lowest relative abundance from top to bottom. The statistical method was Spearman’s rank correlation coefficient. *p < 0.05 and **p < 0.01.

The relationship between the top 10 genera of gut microbiota and concurrent clinical parameters was analyzed (Figures 6A–D). In the Pre group, g_*Fenollaria* was correlated with six clinical parameters: positively with CHE and Hb; negatively with TB, IDB, INR, and MELD-Na (Figure 6A). In the Post1 group, g_*Enterococcus* was negatively correlated with ALT, ALP, TB, and IDB (Figure 6B). The corresponding results of Post2 and Post3 groups are shown in Figures 6C, D.

### Aerobic bacteria increased in the Post1 group

**FIGURE 7:**
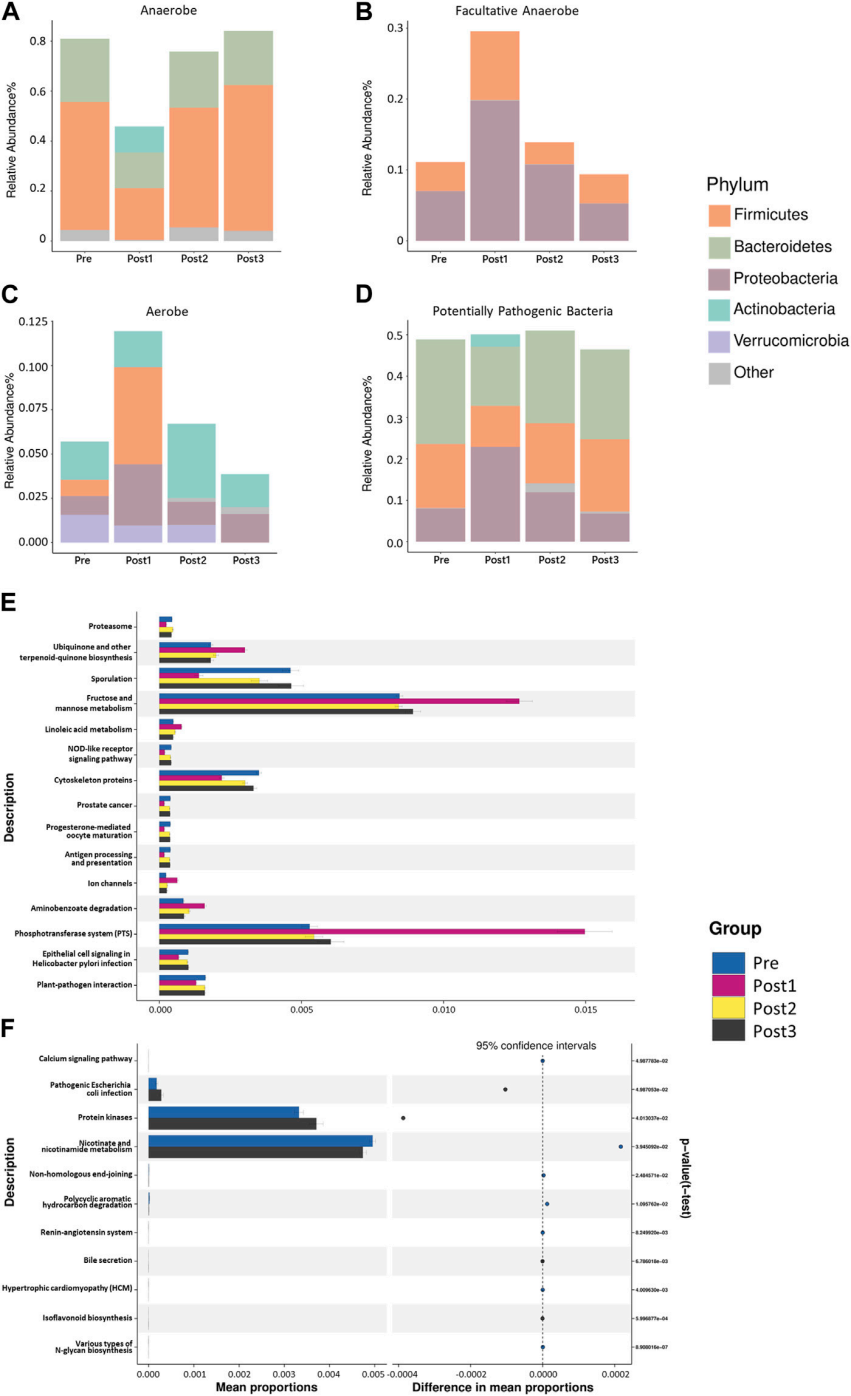
BugBase’s phenotype classification. The phenotypes depicted in **(A–D)** were, in turn, anaerobes **(A)**, facultative anaerobes **(B)**, aerobes **(C)**, and potentially pathogenic bacteria **(D)**. The horizontal axis in the figure represented different groups, the vertical axis represented relative abundance, and different colors represented different phyla. The results shown in **(A, B)** were statistically different (*p* < 0.001). The results shown in **(C,D)** were not statistically different (*p* > 0.05). The statistical method was the Kruskal–Wallis test, with *p* < 0.05. PICRUSt2-based predicted functional analysis. The contrast groups represented by **(E, F)** plots were Pre, Post1, Post2, and Post3 **(E)**; Pre and Post3 groups **(F)**. The significantly different KEGG metabolic pathways were shown at the third level and their proportions among different groups, with different colors representing different groups.

In order to understand the detected bacterial phenotypes, we employed BugBase’s phenotype classification to analyze the bacterial phenotypes (Figures 7A–D). The horizontal axis in the figure represented different groups, the vertical axis represented relative abundance, and a color block area represented a phyla proportion.

As expected, the most notable difference in the bacterial phenotype was found in the Post1 group. Specifically, the relative abundance of anaerobic bacteria was significantly lower in the Post1 group than that in others (*p* < 0.001) (Figure 7A). The reduction of anaerobic bacteria was mainly due to the relative decrease in Firmicutes and Bacteroidetes. The anaerobic type of Actinobacteria was only present in the post1 group, which was also a feature of the gut anaerobic bacteria after LTx.

On the contrary, the relative abundance of facultatively anaerobic bacteria was significantly increased after the surgery (*p* < 0.001) (Figure 7B). The increase in Proteobacteria and Firmicutes contributed to this. The relative abundance of aerobic bacteria in the Post1 group was significantly promoted, which was mainly due to the increase in Firmicutes and Proteobacteria (Figure 7C). The potentially pathogenic differences of the microbiota among the groups exhibited no statistical difference among groups (Figure 7D).

### Predicted functional analysis of LTx recipients’ gut microbiota

In order to analyze the composition of functional genes in the existing sequenced microbial genomes, we used PICRUSt2 from the Kyoto Encyclopedia of Genes and Genomes (KEGG) database and the MetaCyc database to perform KEGG Orthology (KO) analysis to predict the microbial metabolism function (Figures 7E, F). The figure shows the significantly different KEGG metabolic pathways at the third level and their proportions in each groups.

As expected, the Post1 group exhibited the most notable uniqueness compared to the other three groups, showing a decrease in nine pathways and an increase in six pathways (Figure 7E). The most promoted pathway was related to the fructose and mannose metabolism and phosphotransferase system pathways, suggesting that the metabolic function of gut microbiota was significantly affected after LTx.

When we analyze the metabolism difference between the Pre group and Post3 group (Figure 7F), we only found 11 metabolic pathways that were statistically different between the two groups. Then, 6–12 months after transplantation, the gut microbiota exhibited enhanced protein kinases and pathogenic *Escherichia coli* infection and reduced nicotinate and nicotinamide metabolism.

## Discussion

Gut microbiota plays a significant role in the host. It interacts with the host immune system and participates in food digestion, intestinal endocrine regulation, drug action, metabolism, and toxin elimination (Lynch and Pedersen, 2016). The gut microbiota not only affects the local intestines but can also impact distal organs including the liver through the gut–liver axis. In healthy conditions, the gut–liver axis is maintained by the intestinal barrier, which strictly limits the access of gut bacteria and their derivatives into the portal vein circulation. However, the intestinal barrier can be interrupted by local or systemic disease, leading to increased intestinal permeability. As a consequence, gut bacteria and their derivatives have easier access to the portal vein circulation, further reaching the liver (Lau et al., 2023). To better understand the information on gut microbiota of LTx recipients, this study presents a broader and more in-depth profile of the gut microbiota dynamics in adult LTx patients in Northeast China.

The factors that affect gut microbiota are complex and intersecting, such as age, ethnicity, geography, host genetics, gender, and lifestyle factors (Park et al., 2021). Previous studies have shown that region and ethnicity have an impact on human gut microbiota (Mueller et al., 2006; Gaulke and Sharpton, 2018). Different regions and ethnic groups may have different diet habits and lifestyle. A study of 314 healthy volunteers from nine provinces in China showed that regions, ethnic groups, and lifestyles affect the structure of intestinal flora in China (Zhang et al., 2015). All LTx recipients in this study were from Northeast China. Northeast China is located on the northeastern edge of the Asian continent and includes Liaoning, Jilin, Heilongjiang, and the eastern part of Inner Mongolia. In anthropology, the population belongs to the Mongoloid–Manchu–Tungus race. People’s eating habits include a high-fat and high-salt diet, with a preference for rice and pickled fermented foods.

Generally, we found that the LTx recipients’ gut microbiota exhibited significant fluctuation and remarkable resilience by quick recovery during the perioperative period and 1-year follow-up. However, this recovery did not reach the preoperative state. During the recovery of gut microbiota after LTx, there were some adaptive changes in species composition and metabolic pathways. The patients with better healthy conditions before transplantation exhibited a more remarkable reduction in the diversity, but all patients’ bacterial composition profoundly restructured after LTx. In end-stage liver disease patients, higher levels of CHE and Hb are indicative of better clinical status, while higher levels of TB, IDB, INR, and MELD-Na are associated with poorer clinical status. g_*Fenollaria*, belonging to the Bacillota phylum, is positively correlated with better clinical status. It is generally believed to have the capability to produce short-chain fatty acids (Buffet-Bataillon et al., 2022). This study observed, for the first time, the correlation between g_*Fenollaria* and preoperative clinical parameters in LTx patients.

The patients with end-stage liver disease on the waiting list have already shown a significant decrease in gut microbiota diversity compared to healthy individuals (Lai et al., 2022). During the perioperative and follow-up periods, events influence the recipient’s physiological condition, including the surgery, long term high-grade and mixed antibiotic use, postoperative complications, and clinical interventions. As an indicator of gut microbiota balance, we observed the changes in the gut microbiota diversity of LTx recipients. The severe decrease in diversity of the Post1 group may be caused both by the antibiotic exposure and LTx surgery.

Bacteroidetes and Firmicutes phyla comprise approximately 90% of the bacteria in the healthy human gut, and they were related to the energy metabolism; the minority bacteria belong to Proteobacteria, Actinobacteria, Fusobacteria, and Verrucomicrobiota phyla. The Firmicutes phylum contains over 250 genera of bacteria, including *Lactobacillus* and *Clostridium*, and the Bacteroidetes phylum includes around 20 genera, the most abundant being *Bacteroides* (Mariat et al., 2009; Goodrich et al., 2014). The increase in Bacteroidota was observed during weight loss in obese individuals on a vegetarian diet (Ley et al., 2006). Firmicutes break down polysaccharides in food into nutrients to increase the body’s energy utilization, thus contributing to obesity (Turnbaugh et al., 2006; Tagliabracci et al., 2013). In this study, the gut microbiota of patients before LTx was dominated by p_Firmicutes and p_Bacteroidota. The relative abundance of p_Bacteroidota significantly decreased in the Post1 group. Although Firmicutes showed no significant difference in relative abundance, *Enterococcus* in Firmicutes significantly increased in the Post1 group, masking the widespread decrease in the relative abundance of other bacterial genera in Firmicutes, such as *Prevotella*, *Peptoniphilus*, *Streptococcus*, *Finegoldia*, *Veillonella*, *Faecalibacterium*, *Dialister* and *Ruminococcus*.

*Enterococcus* is a potential pathogen in hospital settings because of its survival ability in an antibiotic-rich harsh environment (Krawczyk et al., 2021). The Post1 group exhibited a high abundance of g_*Enterococcus*, possibly due to the use of antibiotics which depleted other bacteria but spared g_*Enterococcus* in the gut shortly after LTx. Although g_*Enterococcus* is not considered a hypervirulent microorganism, several traits associated with it have been linked to diseases. These bacteria are capable of evading the immune system by attaching to the host cells, extracellular matrix, and inert materials, such as a variety of medical devices, and form biofilms that make them resistant to antibiotic killing (Mohamed and Huang, 2007; Maestre et al., 2012). *Enterococcus* can not only cause gastrointestinal tract infection but also, through its translocation, enable infection in sites including the urinary tract, wounded epithelium, heart, and blood (Kao and Kline, 2019). In this study, the Post1 group showed a significant increase in *Enterococcus*, which may highlight the risk of *Enterococcus* as a conditional pathogen causing perioperative infections. Interestingly, other than *Enterococcus*, the potential pathogenicity of the microbiota in the Post1 group was lower than that of the other groups, which may be related to the effective use of antibiotics.

*Prevotella* was a “probiotic” associated with a healthy plant-based diet. In populations with improved glucose metabolism after a healthy diet, the *Prevotella*/*Bacteroides* ratio was higher. Fecal transplantation of *Prevotella* in the mouse model improved their glucose metabolism and liver glycogen content (Kovatcheva-Datchary et al., 2015). The changes in the relative abundance of *Prevotella* after LTx suggest that surgery and antibiotic use may cause metabolic disorder shortly after transplantation.

In a healthy gut, anaerobic bacteria are the most abundant microbiome, and the intestinal dominant bacteria Firmicutes and Bacteroidetes are anaerobes (Goodrich et al., 2014). *Bifidobacterium* and *Akkermansia*, as probiotics, are also anaerobes. The anaerobic bacteria participate in the metabolism of short-chain fatty acids, oligosaccharides, resistant starch, and fiber (Sims et al., 2014; Wu et al., 2014; Andoh, 2016; Hills et al., 2019). In this project, we discovered the relative abundance of anaerobic bacteria decreased significantly in the Post1 group, while both facultative anaerobic and aerobic bacteria were increased. We thought these changes were mainly related to antibiotic use. The postoperative antibiotics usually used were cefoperazone or carbapenems, both of which had the function of inhibiting anaerobic bacteria. Antibiotics could directly or indirectly disrupt the gut barrier (Tulstrup et al., 2015; Shi, et al., 2018), which also interfered with an intestinal anaerobic environment.

KEGG analysis revealed a decrease in sporulation and cytoskeleton protein pathways in the Post1 group, which indicated a decline in microbial activity. The increase in fructose and mannose metabolism was correlated with the increase in serum uric acid levels in gout patients (Chu et al., 2021) and may facilitate infections such as *Klebsiella* (Horng et al., 2018) to deteriorate dysbiosis.

After 1 year of transplantation, we found that although the end-stage liver disease was released, the diversity of gut microbiota was still significantly lower than that in the Pre group, which may be caused by the long-term use of immunosuppressants. Previous studies have found that the immunosuppressants affect the gut microbiota in the LTx rat model, and this change was positively correlated with the dose (Jiang et al., 2018).

Comparing the differential species among the Pre, Post2, and Post3 groups, the increased relative abundance of two clinically common pathogenic bacteria, *Escherichia–Shigella* and *Klebsiella*, was noteworthy. *Escherichia*–*Shigella* and *Klebsiella* were significantly elevated in aged sepsis-related liver injury rats (Liang et al., 2022). *Escherichia–Shigella* has been linked to obesity-related diseases such as NAFLD (Xin et al., 2022). *Klebsiella* was a well-known pathogen causing hospital-acquired infections, which has been increasingly observed worldwide as the causative agent of liver abscesses (Siu et al., 2012). In this study, the increase in *Escherichia–Shigella* and *Klebsiella* provides us with a reference for understanding the replacement of pathogenic bacteria in transplant recipients at different time windows after surgery.

*Acidaminococcus* was the only species that was elevated in the Post3 group in the top 10 differentially abundant genera. *Acidaminococcus* is a genus of p_Firmicutes. Previous studies have shown that the increased relative abundance of *Acidaminococcus* was related to constipation curing (Tian et al., 2020.), occurrence of obesity (Kaplan et al., 2019; Osborne et al., 2020), and negative correlation with high ALT levels in HBsAg-positive individuals (Yun et al., 2019). It was suggested that the gut microbiome may evolve to favor the host to adopt the post-LTx physical condition as the improvement of the patient’s nutritional status and the end-stage liver disease symptom (ascites, tissue edema, and virus infection) were relieved, although the average BMI is not elevated.

Other than *Acidaminococcus*, the reciprocal changes in *Faecalibacterium* and *Veillonella* were observed after LTx during the 6–12-month period . Decreased *Faecalibacterium* and increased *Veillonella* relative abundance were observed under autoimmune hepatitis (Liwinski et al., 2020). Our result suggests the relief of liver disease and the improved healthy condition.

*Lactobacillus* and *Akkermansia* were well-recognized probiotics (Del Pozo, 2018; Depommier et al., 2019; Saeedi et al., 2020), but the relative abundance of these two probiotics gradually decreased during the follow-up of LTx recipients, providing evidence for supplementation of probiotics and that prebiotics may be beneficial.

At the end of the follow-up, the gut microbiota of LTx recipients exhibited activity after 6–12 months of recovery. The changes in nicotinate and nicotinamide metabolism and protein kinases were the most prominent compared with the Pre group. Previous studies have shown that vitamin B group deficiency occurred in patients with liver disease: vitamin B1 and B6 reduced in patients with liver disease (Henderson et al., 1986; Boo, 2021; Kummen et al., 2021), and vitamin B12 deficiency is an independent predictor of the severity of NASH histology in terms of disease activity and fibrosis grade (Mahamid et al., 2018). Gut microbiota in PSC and IBD patients showed higher levels of metabolic pathways related to vitamin B than healthy controls (Kummen et al., 2021). We found that, compared to pre-operation, after 6–12 months of recovery, the LTx patients showed a decrease in the nicotinate and nicotinamide metabolism pathway, which reflect the alteration of vitamin B metabolism. The use of immunosuppressants impacts the gut microbiota by causing susceptibility to infection, reflected by an enhanced pathogenic *E. coli* infection pathway. Healthy population is lacking in this study, and we cannot determine if the Pre and Post3 groups are distinct with the healthy controls.

## Conclusion

**FIGURE 8:**
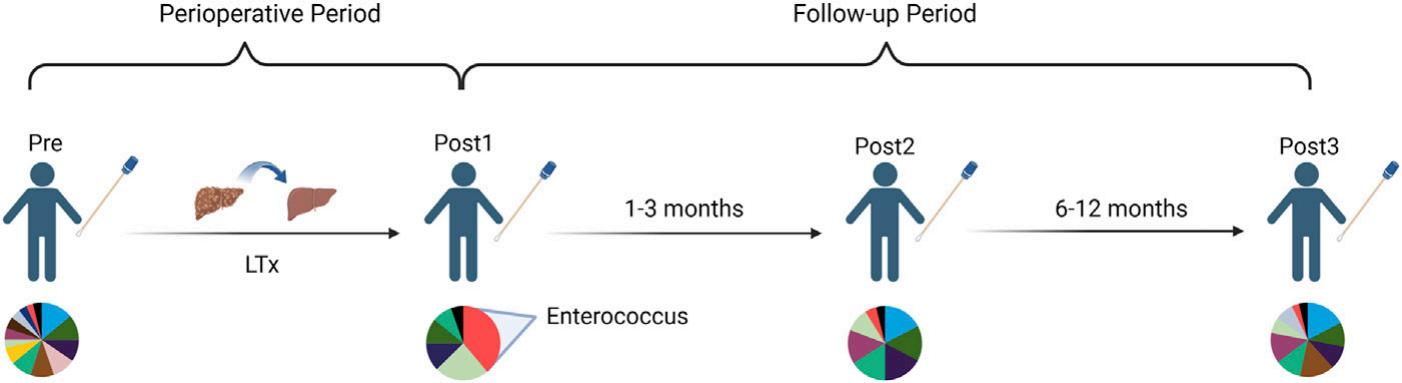
Schematic diagram of a longitudinal comparison of gut microbiota changes in LTx recipients before and 1 year after LTx.

We conducted a comprehensive investigation of the diversity, relative abundance, compositional variation, phenotype, and metabolic function of the gut microbiota of LTx recipients during the perioperative period and 1-year follow-up period. We found the gut microbiota of LTx recipients undergoes significant changes during the perioperative period after LTx. The microbiota rapidly recovers during the first 3 months, and after 1 year of recovery, it approaches but remains distinct from the preoperative state, which exhibited an alteration to adopt the post-LTx condition (Figure 8).

## Data Availability

The datasets presented in this study can be found in online repositories. The names of the repository/repositories and accession number(s) can be found in the article/Supplementary Material.
