# Supplementary material for: Gut microbiota dynamics in a 1-year follow-up after adult liver transplantation in Northeast China
Source: Front Physiol. 2023 Dec 22;14:1266635. doi: 10.3389/fphys.2023.1266635 (PMC10766776; doi:10.3389/fphys.2023.1266635)
Supplement: Supplementary file 4 [file DataSheet1.docx]

Supplementary Material

Gut microbiota dynamics in a 1-year follow-up after adult liver transplantation in Northeast China

Ruoyan Zhang , Wei Qiu , Xiaodong Sun , Jing Li , Xiaochen Geng , Shichao Yu , Ying Liu , Heyu Huang , Mingyue Li , Zhongqi Fan , Mingqian Li *, Guoyue Lv *

*** Correspondence:** Guoyue Lv: [lvgy@jlu.edu.cn](mailto:lvgy@jlu.edu.cn); Mingqian Li: [mingqianli@jlu.edu.cn](mailto:lvgy@jlu.edu.cn)

##
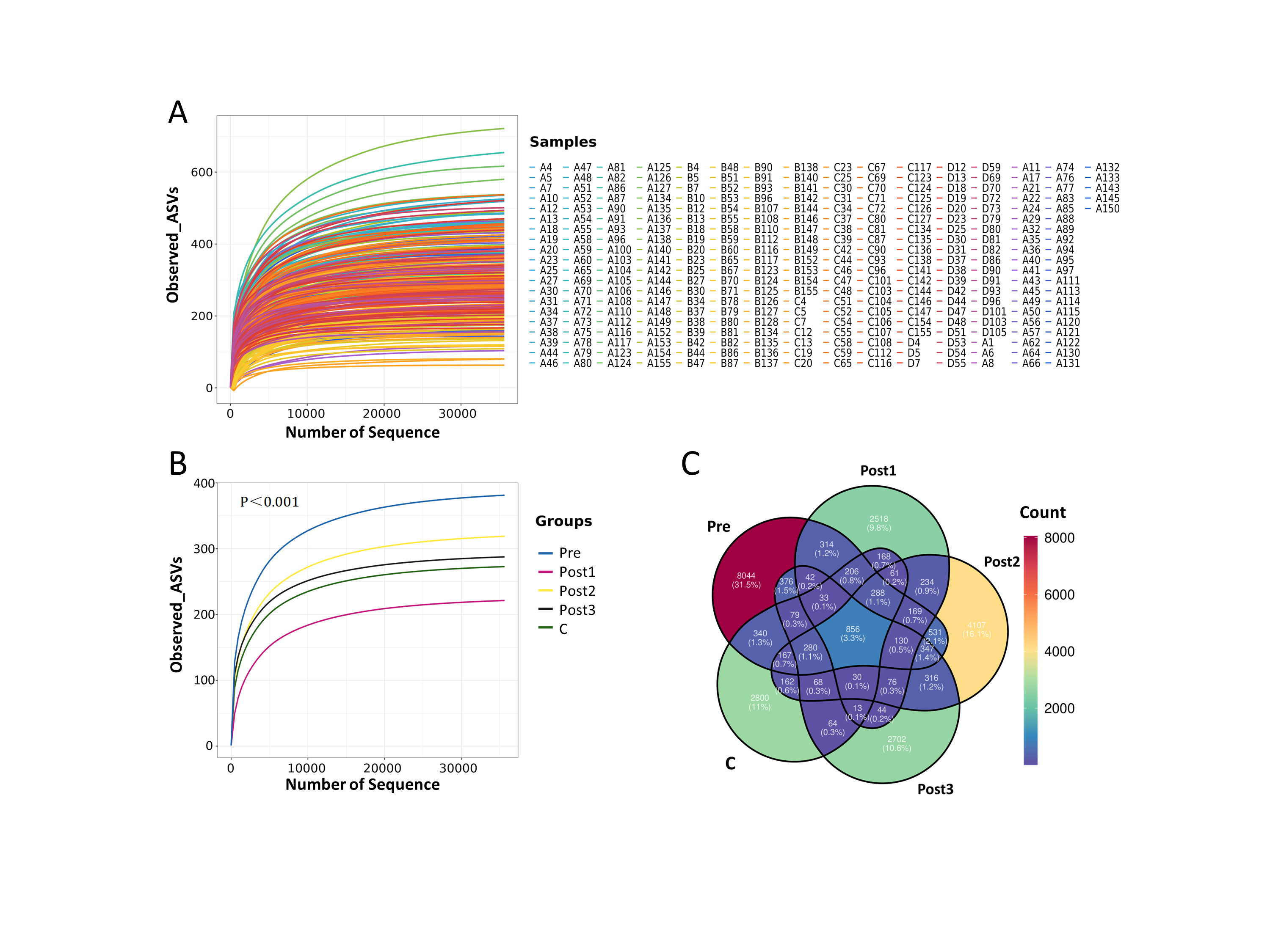
Supplementary Figures

**Supplementary Figure S1.**  Rarefaction curve and venn diagram. Rarefaction curves and venn diagram were plotted at the ASVs level. (A)The rarefaction curves were plotted according to the samples. In the sample number, "A", "B", "C" and "D" represents Pre group, Post1 group, Post2 group and Post3 group, respectively. The numbers represent patient ID. The correspondence between the specimen names in article, the original sample numbers and the group names was shown in Supplementary Table S2. (B) The rarefaction curves were plotted according to the groups were shown for Pre, Post1, Post2, Post3 and C. (C) Venn diagram shown the distribution of ASVs obtained in each group.


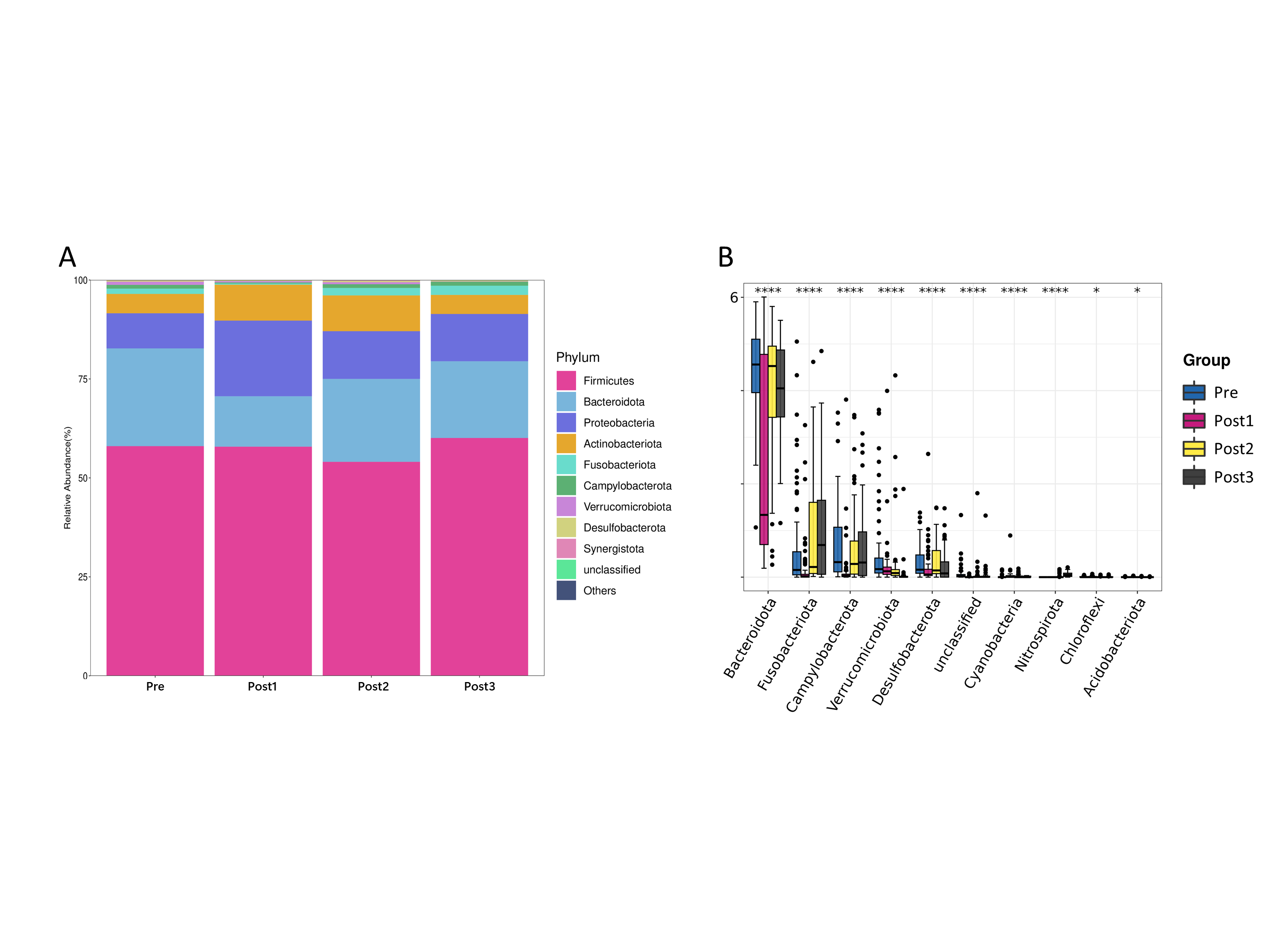


**Supplementary Figure S2.** (A) The relative abundance distribution of top 10 species at the phylum level in each group. (B) Taxa with statistically significant differences at the phylum level in each group in order of relative abundance from high to low. Kruskal-Wallis test was used to determine the statistical significance. **P*<0.05, ***P*<0.01, ****P*<0.001, and *****P*<0.0001.


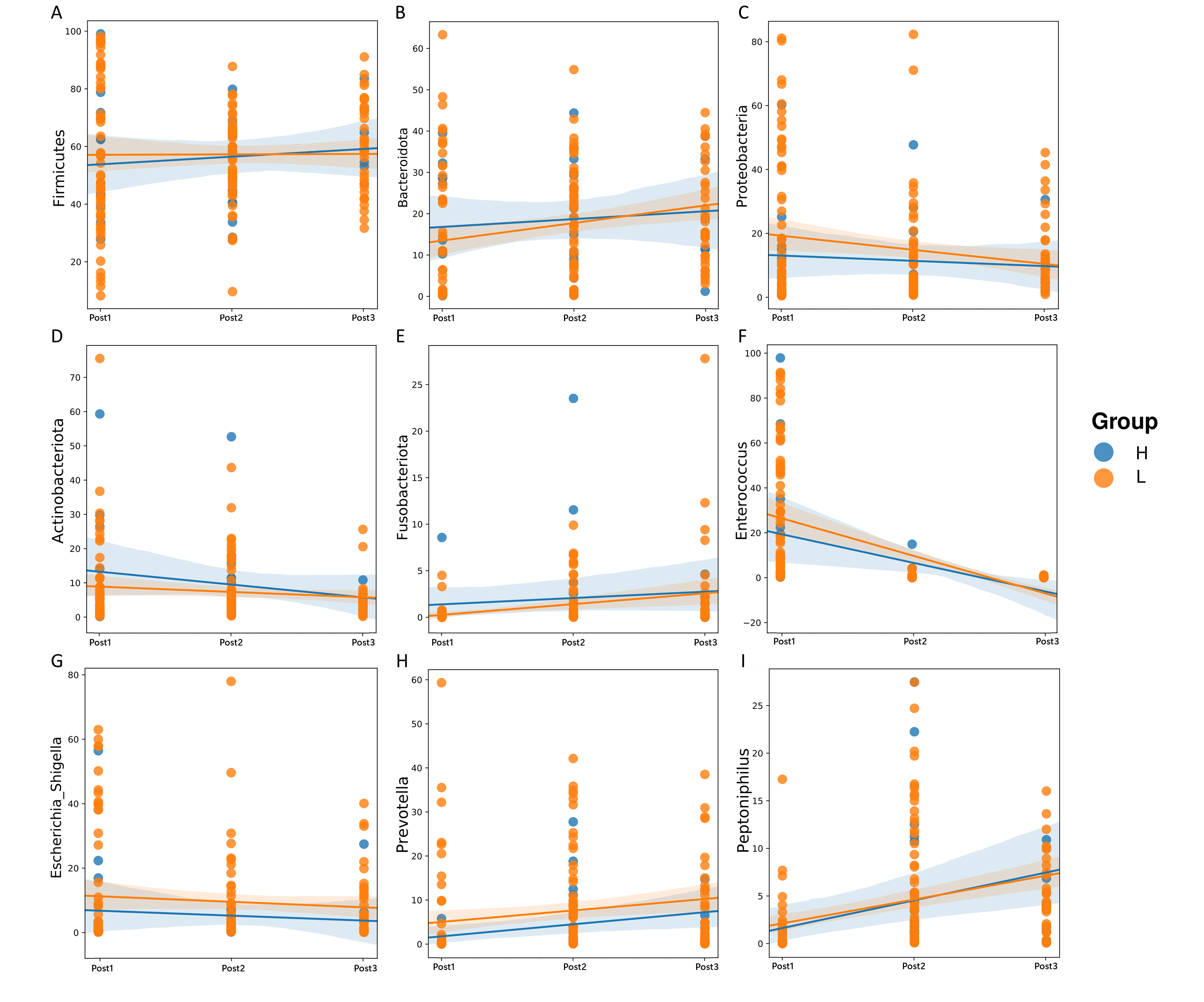


**Supplementary Figure S3.**  The correlation between MELD-Na score and gut microbiota. Using MELD-Na≥20 as a threshold, it was divided into H and L groups. The top 5 relative abundance phylum and genus were selected from high to low to analyze the impact of MELD-Na on each one Using Linear mixing model. (A-E) The results showed that there were no statistically difference in the impact of MELD-Na on p_Firmicutes (*P*=0.538), p_Bacteroidota (*P*=0.153), p_Proteobacteria (*P*=0.348), p_Actinobacteriota (*P*=0.292), and p_Fusobacteriota (*P*=0.334). (F-I) The results showed that there was no statistically significant difference in the impact of MELD-Na on g_Enterococcus (*P*=0.298), g_Escherichia-Shigella (*P*=0.552), g_Prevotella (*P*=0.568), g_Peptoniphilus (*P*=0.753). The statistical method was ANOVA analysis, with *P*<0.05.
